# Rapid Quantification of 3D Collagen Fiber Alignment and Fiber Intersection Correlations with High Sensitivity

**DOI:** 10.1371/journal.pone.0131814

**Published:** 2015-07-09

**Authors:** Meng Sun, Alexander B. Bloom, Muhammad H. Zaman

**Affiliations:** 1 Department of Biomedical Engineering, Boston University, Boston, Massachusetts, United States of America; 2 Department of Molecular Biology, Cellular Biology and Biochemistry, Boston University, Boston, Massachusetts, United States of America; 3 Howard Hughes Medical Institute, Boston, Massachusetts, United States of America; Universidad Carlos III of Madrid, SPAIN

## Abstract

Metastatic cancers aggressively reorganize collagen in their microenvironment. For example, radially orientated collagen fibers have been observed surrounding tumor cell clusters *in vivo*. The degree of fiber alignment, as a consequence of this remodeling, has often been difficult to quantify. In this paper, we present an easy to implement algorithm for accurate detection of collagen fiber orientation in a rapid pixel-wise manner. This algorithm quantifies the alignment of both computer generated and actual collagen fiber networks of varying degrees of alignment within 5°°. We also present an alternative easy method to calculate the alignment index directly from the standard deviation of fiber orientation. Using this quantitative method for determining collagen alignment, we demonstrate that the number of collagen fiber intersections has a negative correlation with the degree of fiber alignment. This decrease in intersections of aligned fibers could explain why cells move more rapidly along aligned fibers than unaligned fibers, as previously reported. Overall, our paper provides an easier, more quantitative and quicker way to quantify fiber orientation and alignment, and presents a platform in studying effects of matrix and cellular properties on fiber alignment in complex 3D environments.

## Introduction

The majority of cancer related deaths are not caused by primary tumors but instead by secondary sites where cancer cells have metastasized [1]. Cancer cell metastasis requires degradation and remodeling of the surrounding extracellular matrix (ECM), cellular loss of anoikis signaling, and the cells to undergo epithelial to mesenchymal transition (EMT) [2]. While numerous studies have emphasized the signaling pathways involving anoikis resistance and EMT, similar emphasis has not been placed on quantitative study of ECM degradation and remodeling [3–5]. *In vivo*, the ECM surrounding cells is composed of polysaccharides and proteins [6]. Though the exact composition of ECM is tissue dependent, its primary structural component is collagen, a stiff triple-helical protein that self assembles into load-bearing fibers. Over 30% of all protein in the human body consists of collagen, and roughly 90% of this collagen is type I collagen [7].

While collagen density has been studied extensively in regard to cancer invasiveness [8] and diffusion of therapeutic molecules [9,10], quantitative methods for determining collagen fiber orientation have largely been ignored. Traction forces from cells are primarily responsible for changing collagen fiber orientation [11,12], as seen in wound healing [13,14]. Collagen orientation in turn guides cell migration and polarization [15]. *In vitro*, mechanical strain [16] or microfluidic channels [17] also influence the collagen fiber orientation. Radially orientated collagen fibers have been observed surrounding tumor cell clusters *in vivo* and have been classified as tumor associated collagen signature type 3 [3,5,18]. This potential quantitative image-based biomarker of collagen *in vivo* offers a new prognostic indicator for cancer invasiveness, especially in triple negative breast cancer [19]. However this method only identified a cursory link between the degree of collagen alignment and cancer metastasis, and quantitative tools have not yet been thoroughly established. The quantification of collagen fiber alignment is not only important in studying cancer invasiveness, but also plays a significant role in tissue engineering [20,21].

Nawroth et al. [20] utilized a biometric fingerprint algorithm to successfully identify actin fiber alignment in the muscle cells of the engineered jellyfish. Building upon this biometric fingerprint algorithm, the fiber alignment quantification method developed in this paper uses the squared gradient vector to enhance the accuracy of the pixel-by-pixel orientation calculation [22]. Previous approaches of measuring collagen alignment have mainly used image analysis tools. Kim et al. [23] pioneered using the fast Fourier transform (FFT) to calculate the relative orientation intensity in frequency domain, and Pang et al. [24,25] applied the FFT in their work as well. Riching et al. [16], Eliceiri et al. [19] and Bredfeldt et al. [26] used the Curvelet transform [27] to calculate collagen fiber network orientation. Both FFT and Curvelet transform give the relative orientation distribution of the whole image rather than individual pixel orientation information. Daniels et al. [21], Vader et al. [28] and Abhilash et al. [12] calculated the principal curvature directions of each pixel within an image from Hessian matrices. Hessian matrices give detailed and accurate orientation estimations, however they are computationally expensive due to the solving process of the eigenvalue and eigenvector of the matrices at each pixel. Karlon et al. [29] and Kaunas et al. [30] used a local first-order intensity gradient to quantify orientation. Though this method is relatively fast, due to an increased sensitivity to noise it is less accurate in comparison with the Hessian matrices method.

In addition to quantification of fiber orientation, there are several ways of quantifying the degree of alignment from orientation data. These include using only the standard deviation of orientation [3], or determining the ratio of the standard deviation over the orientation distribution width [31]. Some *in vitro* studies also used the alignment index (AI) [12,24,28] to describe the fiber alignment. This alignment index averages the orientation data and originates from the nematic order parameter in liquid crystal theory to determine ordering status of molecules in nematic phase [32,33].

In order to develop an algorithm that is able to quantify fiber orientation in a computationally efficient manner and determine the degree of alignment, we propose a new method in this paper. Here, we present a strategy of quantifying the degree of collagen fiber alignment with a high degree of sensitivity, to allow for generalization of the alignment information and use the scheme to study the correlation between the degree of alignment and other key physical features of the collagen network, including the number of fiber intersections. The algorithm described in this paper is capable of determining the degree of alignment of both unaligned and aligned collagen fibers. We also describe an easy and accurate AI estimation method utilizing only the standard deviation of the fiber network orientation. To validate the algorithm, artificial images mimicking collagen fibers with varying degrees of alignment were generated. Kolmogorov-Smirnov statistics were utilized as a metric for algorithm accuracy [34]. Finally, the quantification tools described in this paper provide a platform for studying different effectors of collagen alignment quantitatively. In the future effectors up regulating or down regulating the degree of ECM alignment, such as matrix metalloproteinases and cell contractile forces, can be quantitatively modeled with these quantification tools.

## Materials and Methods

### Collagen Gel Preparation

To acquire images of collagen fibers Type I collagen gels were made as per previous literature [8]. Type I collagen from rat tail (BD Biosciences, San Jose, CA) was added to an equal volume of 1x neutralizing solution (100 mM HEPES buffer in 2x phosphate buffered saline, pH 7.3). Final concentration of the collagen gels ranged from 2 mg/ml to 4 mg/ml. 1ml gels were allowed to polymerize in 35 mm glass bottom dishes (MatTek, Ashland, MA) at 37°C and 5% CO_2_ for 60 minutes after which 2 ml of 10% v/v FBS-supplemented media was added.

### Spheroid Preparation

MDA-MB-231 cells grown on tissue culture flasks were trypsonized and counted using a hemacytometer. 100ul of media containing 10,000 cells were plated on solidified 1.5% (w/v) agarose. The agarose provides a non-adherent surface allowing the cells to aggregate over 72 hours at 37°C and 5% CO_2_. To generate aligned collagen fibers two spheroids were embedded within close proximity to each other [4]. Two of the resulting spheroids generated on agarose were embedded, via pipetting, into a collagen gel prior to the gels polymerizing.

### Confocal Reflectance Microscopy

To visualize the fiber microstructure of the collagen gels, confocal reflectance microscopy was performed using a scanning confocal microscope (Olympus FV1000) with a 60x 1.2 N.A. water immersion lens. The collagen gels were excited with 488 nm laser, and light between 485 nm and 495 nm was collected. To avoid edge effects, images were acquired at least 100 *μm* into the gel. For acellular collagen gels (gels containing no cells), four 30 *μm* stacks with 0.5 *μm*-thick slices were obtained from randomly selected regions in the gel. For images of aligned collagen fibers, gels containing two embedded spheroids were used and a region of highly aligned collagen, between the two spheroids, was selected for imaging with stack dimensions identical to those of acellular collagen gels. Collagen type I fibrils can range in diameter from 20 nm to several hundred nm [35] while fibers are larger in diameter. Given that the size of each pixel is 0.414 *μm* it is not possible to distinguish fibrils and fibers [36]. When discussing intersections we use the term “fibers” as including both fibrils and fibers.

### Generating Fiber-like *In Silico* Images

**Fig 1:**
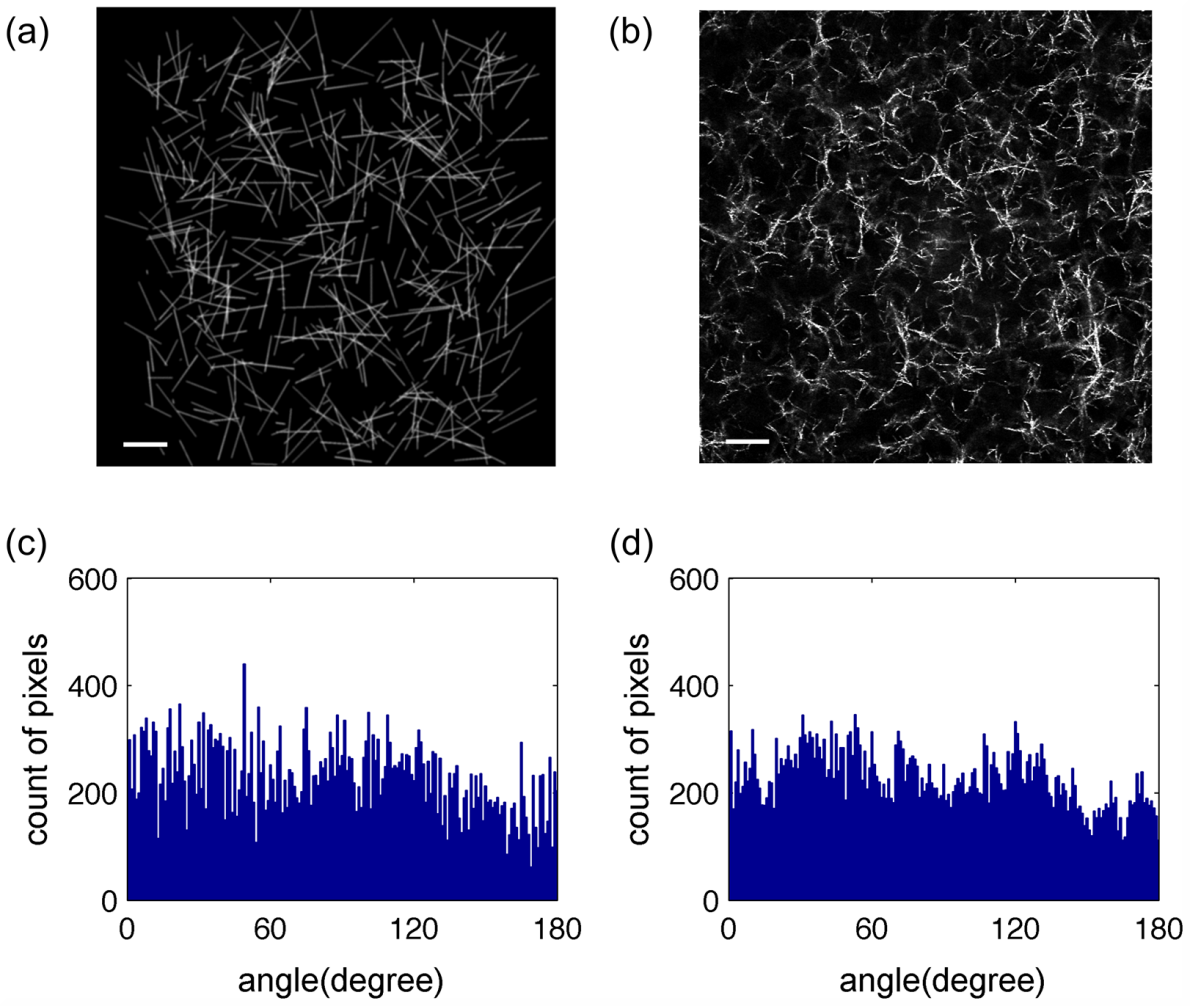
Computer-generated fiber images validate the orientation detection algorithm. The computer-generated random fiber network (a) mimicked the actual acellular collagen gel fiber network at 2 mg/ml (b). The scale bars are 20 *μm*. Histograms of the theoretical (c) and the algorithm determined (d) orientation values of the computer-generated fiber network (a) were compared using KS statistics.

To test the accuracy of the collagen orientation calculations, computer-generated images mimicking real collagen fiber gel networks, with varying degrees of alignment, were created in Matlab (R2009b, MathWorks, Inc., US). The ‘fibers’ were initially created as straight-line segments, as each fiber was assumed to be straight, even though real collagen fibers can have both non-linear and linear domains [37]. The generated images served to validate the orientation algorithm at each pixel and the fiber intersection detection algorithm. Whether the fiber was nonlinear or straight did not affect the validation efficiency significantly, because in the actual collagen images the fibers are linear locally. The length of the fiber was assumed to have a Gaussian distribution, with the mean and standard deviation values obtained from the literature [8]. The width of the artificial fibers was achieved by blurring the images with Gaussian filter kernel convolution, whose sigma value corresponded to the mean value of fiber width found in the literature [8]. Adding Poisson noise mimicked white noise due to the microscope [21]. Different numbers of fibers were created to have the fiber fraction range from roughly 16% to 61%. The orientations of aligned collagen ‘fibers’ were assigned a Gaussian distribution with varying standard deviations (*θ*) to mimic different degrees of alignment. A random mode (predominant) orientation was assigned. Random collagen ‘fibers’ were simply assigned to have random orientations. The fiber-wise orientations with the same distribution may have slightly different pixel-wise orientation, due to differences in fiber length. Thus images with pixel-wise standard deviation *θ* ± 0.5° were used for validation in aligned collagen ‘fibers’. For each degree of alignment, 15 image samples were generated (Fig 1). To determine if different distributions of orientations influence either the algorithm or AI, ‘fibers’ with Gaussian and Von Mises distributions were generated for validation.

### Image Analysis

Each individual frame within the image stacks of acellular and spheroid embedded collagen gels was analyzed using our algorithm developed in Matlab (R2009b, MathWorks, Inc., US). The algorithm first calculated the collagen orientation at each pixel, building upon the biometric fingerprint algorithm (Peter Kovesi, School of Computer Science & Software Engineering, University of Western Australia) [38]. The orientation estimation algorithm, originating from Hong et al. [22] and Rao et al. [39], calculated the orientation vector at each pixel (*i*, *j*) by the squared x and y components of gradients:

$$J_{x}(i,j)=G_{x}^{2}(i,j)-G_{y}^{2}(i,j),$$

$$J_{y}(i,j)=2G_{x}(i,j)G_{y}(i,j),$$

where *G*_*x*_ and *G*_*y*_ are the gradient vectors in the x and y directions calculated via convolution with the gradient of Gaussian function with a sigma value of 1 and size 7 x 7 pixels. The gradient vectors were smoothed by a second Gaussian filter with a sigma value of 2 and size 13 x 13 pixels to optimize the performance of the algorithm. The orientation was given by [22,39,40]

$$\theta (i,j)=\frac{1}{2}{tan}^{-1}(\frac{J_{y}(i,j)}{J_{x}(i,j)}).$$

Here a four-quadrant inverse tangent function was used resulting in orientations ranging from 0° to 180°. This squared gradient method enhances the orientation detection, as the same orientation gradient vector is reinforced by its opposite direction gradient vector. Both this method and the Hessian matrix method calculated the orientation from enhanced forms of gradients, thus these methods have higher accuracy in orientation estimation compared to the pixel gradient method described in the introduction. Intrinsically, compared with the Hessian matrix method, the algorithm described in this paper avoids the eigenvector and eigenvalues solving process. Furthermore the algorithm is relatively easy and quick in terms of only using Gaussian filters and some simple math on trigonometric functions to obtain pixel-wise orientation data. After the orientation estimation at each pixel, a mask filtered each pixel based on a fiber intensity threshold to identify the collagen fraction of the image. The intensity threshold was considered optimized when nearly all collagen fibers visualized in the images were identified by the algorithm with minimal background noise. The fiber fraction calculated in acellular collagen gels, using this threshold, was similar to previous data at the same fiber concentration [8]. A histogram of the orientations of pixels was plotted to visualize pixel-wise orientation distribution. The error of algorithm was calculated on artificial fiber-like images with varying degrees of alignment. With orientation calculated at each pixel, the alignment index (AI) [24] was calculated to provide a quantitative metric of the degree of alignment of collagen fibers:

$$AI=\left|\frac{1}{N}\overset{N}{\underset{i=1}{\sum }}\left(2cos^{2}\left(\theta_{i}-\theta_{th}\right)-1\right)\right|,$$

where *θ*_*th*_ is the mean orientation angle among the collagen fibers and N is the total number of fiber fraction pixels counted. If collagen fibers are all randomized, AI = 0, whereas all in the same direction, AI = 1.

The number of collagen fiber intersections in each individual frame within the image stack, as an effect of the alignment, was quantified by a second algorithm using the Matlab morphological functions [41–44] as follows: Intersection identification relied on the enhancement of the collagen fiber structure [45,46]. A 2x2 pixel Wiener filter was first applied to remove noise within the images. A threshold of intensity was applied to distinguish the fibers from background noise, as performed in the orientation detection algorithm. A diagonal fill function then connected pixels that were diagonally adjacent but had no orthogonally adjacent pixels. The algorithm also deleted all isolated pixels and most small spurs on the fibers. An interior fill was applied, filling in isolated interior pixels, and were followed by a skeletonization process. A second small spur deletion followed by diagonal filling optimized the results. Fiber intersections were identified at pixels that have more than two branches, and multiple intersections within five pixels were considered a single intersection.

This intersection quantification algorithm was also validated on generated fiber-like images to determine the accuracy of the intersection detection. For this purpose, in the fiber-like artificial images, the theoretical number of intersections was determined using linear geometry properties: if the endpoints of one linear ‘fiber’ are separated by a second linear ‘fiber’ then these two ‘fibers’ intersect and the algorithm calculates the exact location of this intersection. When multiple intersections occur within five pixels only one intersection is counted. The percent error between the theoretical and the estimated number of intersections was calculated to indicate the accuracy of the algorithm.

### Statistical Analysis

To demonstrate that the fiber pixel orientation estimation accurately describes the fiber orientation within the large orientation data set (N ~ 65,000) Kolmogorov–Smirnov (KS) statistics [34] were used. This provided a natural measurement to indicate impurities of algorithm, based on the images generated to mimic the collagen fibers images, i.e., the impurity between the theoretical and the estimated orientations. We cannot perform hypothesis tests based on these KS statistics because a large data virtually guarantees a statistically significant difference. KS statistics identify the maximum difference between the theoretical and the estimated cumulative distribution functions (CDF). This maximum difference was used to estimate the error in the orientation detection algorithm [34].

For testing any significant differences between conditions, Welch’s two sample t-test in R [47] (Vienna, Austria) was used. The correlation between the degree of alignment and number of collagen fiber intersections was studied also using R [47]. Using the maximum likelihood multi-linear regression (MLMLR) the effects of changing fiber fraction and of the degree of alignment on the intersections were separately studied. Both the ANOVA test and the 95% confidence intervals of the regression coefficients show the significance of the dependence and correlation. The residuals of the MLMLR model also have a normal distribution, without changing the distribution patterns along the change of the degree of alignment (AI) or fiber fraction, showing that the model selection is efficient [48], [34].

## Results

The orientation algorithm was tested initially on computer-generated images designed to mimic acellular collagen gels. Varying the degree of alignment verified that the quantitative method developed in this paper was able to distinguish small differences in the degree of alignment. The images of acellular collagen gels and spheroid embedded collagen gels were investigated to study their alignment features. With these tools accurately quantifying the fiber degree of alignment, a negative correlation was found between the number of fiber intersections and the degree of alignment.

**Fig 2:**
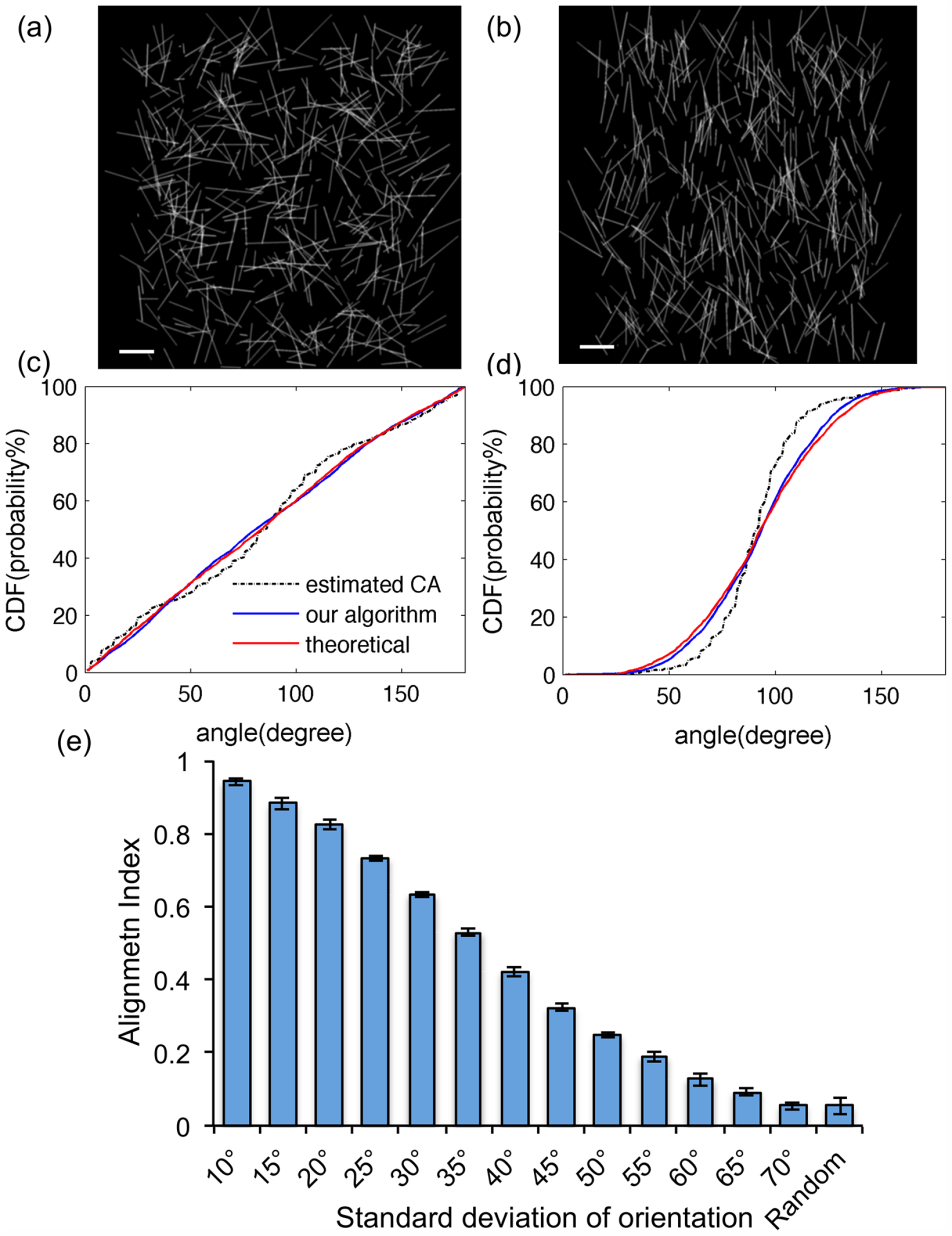
Analysis of computer-generated images with differing alignments validates the accuracy of the algorithm. (a) Computer-generated randomly distributed collagen fibers mimicked the acellular network at 2 mg/ml. (b) Computer-generated Gaussian distributed collagen fibers mimicked highly aligned collagen fiber networks at 2 mg/ml. The scale bars are 20 *μm*. (c) and (d): KS plots of (a) and (b) showed that the algorithm determined fiber orientations are very similar to the theoretical fiber orientation. The dashed lines are the CDFs of estimated orientations obtained from the CurveAlign algorithm as comparisons. (e) The resulting AI values, as determined by the algorithm, reflected the standard deviation of the computer-generated fiber orientations with high sensitivity. With the Welch two sample t-test P < 0.05, all pairs are significantly different except between 70° and random. Thus, the algorithm is able to distinguish the alignment index of two networks whose standard deviations differ by only 5°.

The artificial images were used to study the accuracy in quantifying the degree of alignment and to determine the sensitivity of our method in distinguishing subtle differences in the degree of alignments within a large range of fiber fraction. Both the random (Fig 2A) and aligned computer-generated fibers (Fig 2B) have very similar orientation CDF when comparing the calculated and theoretical values (Fig 2C and Fig 2D). The difference of CDF averaged roughly about 5% among varying degrees of alignment in AI ranging 0 to 0.94 (Table 1), thus the orientation algorithm is accurate throughout the range of the degrees of alignment tested. Furthermore, the algorithm is able to detect a change in the standard deviation of orientation of 5° (Fig 2E). When AI < 0.16, there are no significant differences between the estimated AI of images with fiber fractions of 16% (2mg/ml), 27% (3mg/ml) and 35% (4mg/ml) with the same degree of alignment. When AI > 0.16 there are significant differences in the estimated AI, but at most differ by only ~ 0.02. Von Mises distribution also gave a similar error of algorithm (data not shown).

**Table 1:** The AI values change according to the standard deviation of fiber orientations in computer-generated collagen networks.

Pixel-wise standard deviation (°)	Average estimated AI at 16% fiber fraction*	Error of algorithm at 16% fiber fraction (%)	Error of algorithm at 27% fiber fraction (%)	Error of algorithm at 61% fiber fraction (%)
5	0.98	17.93±6.04	16.85±7.11	19.61±5.13
10	0.94	7.83±3.52	7.05±2.61	7.99±2.32
15	0.88	6.81±2.63	6.20±2.57	5.70±2.15
20	0.83	5.77±2.25	6.69±1.15	6.96±1.58
25	0.73	6.25±1.38	7.78±1.95	9.68±2.41
30	0.63	5.56±1.67	6.46±1.32	8.92±2.46
35	0.53	4.59±1.06	5.61±1.29	8.30±2.35
40	0.42	4.12±1.30	5.62±1.76	6.65±1.89
45	0.32	3.36±0.89	4.50±0.94	5.37±1.13
50	0.25	3.21±0.78	3.70±1.21	4.36±0.93
55	0.19	2.90±0.97	2.93±0.37	3.64±0.85
60	0.13	2.38±0.49	2.68±0.70	2.55±0.59
65	0.09	2.44±0.39	2.58±0.54	2.39±0.99
70	0.06	2.73±0.66	2.19±0.47	1.89±0.53
Random	0.05	2.50±0.50	2.30±0.53	1.49±0.52

The accuracy of orientation detection was calculated by determining the average value of the maximum CDF difference of 15 computer-generated images for each standard deviation condition. The error of the algorithm was quantified by the average maximum absolute difference between the theoretical and estimated CDF values for 15 images. The error range is obtained by the standard deviation of maximum CDF difference within the 15 images. Computer generated images of increasing fiber fraction were used until errors > 10% were detected when AI < 0.94. The algorithm more accurately calculates ‘fiber’ orientation when the ‘fibers’ are less aligned. The average error of the algorithm (4.32%) in the range of AI ~ [0,0.94] leads to an average error in AI ~ 0.029. The larger errors when AI ~ [0.94,1] may be due to fiber overlap of the highly aligned fibers.

* The standard deviations of the estimated AI are at most 0.02 within 15 samples.

As a comparison, another collagen fiber network orientation quantification algorithm CurveAlign [19,26,27,49] based on Curvelet transform theory was also tested on the same set of generated fiber-like images. Within Matlab, our method takes approximately 0.588 seconds (CPU time = 0.730) while the NewCurve function, which calculates the orientation data in the CurveAlign algorithm takes approximately 1.409 seconds (CPU time = 1.680) to process each image. The CDF functions of the CurveAlign estimated results were also presented in the condition of different alignments (Fig 2C and Fig 2D). The accuracy of our algorithm is as good as the CurveAlign algorithm.

**Fig 3:**
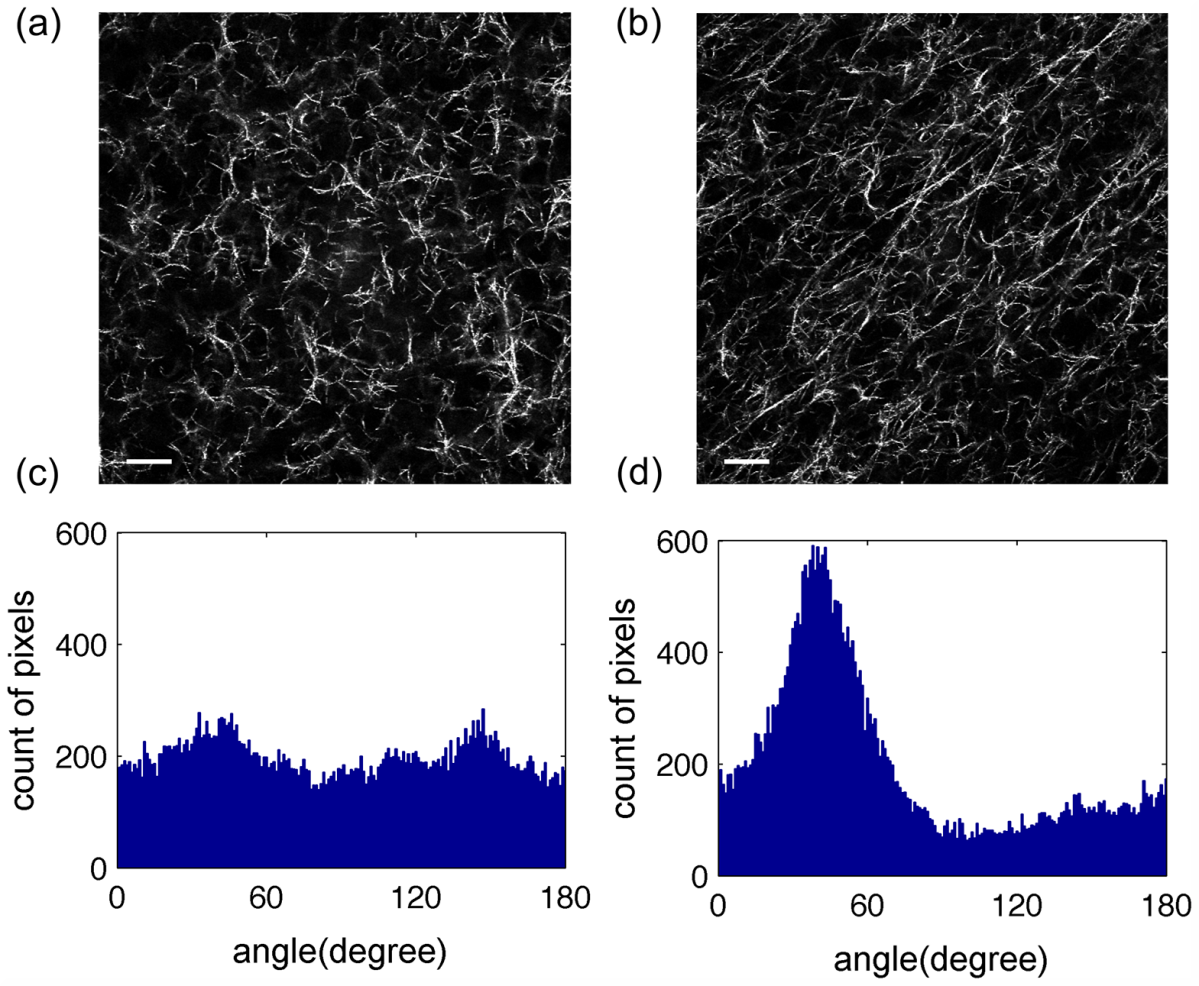
The spheroid embedded collagen gels at 2 mg/ml had an apparent peak in pixel-wise orientation distributions. Images of acellular (a) and spheroid embedded (b) 2 mg/ml collagen gels were analyzed using the orientation detection algorithm. The scale bars are 20 *μm*. The orientation histogram of the acellular collagen gel (c) appeared to have a random distribution (AI = 0.031) while the orientation histogram of the spheroid embedded collagen gel (d) appeared to have an apparent mode orientation (AI = 0.416).

**Fig 4:**
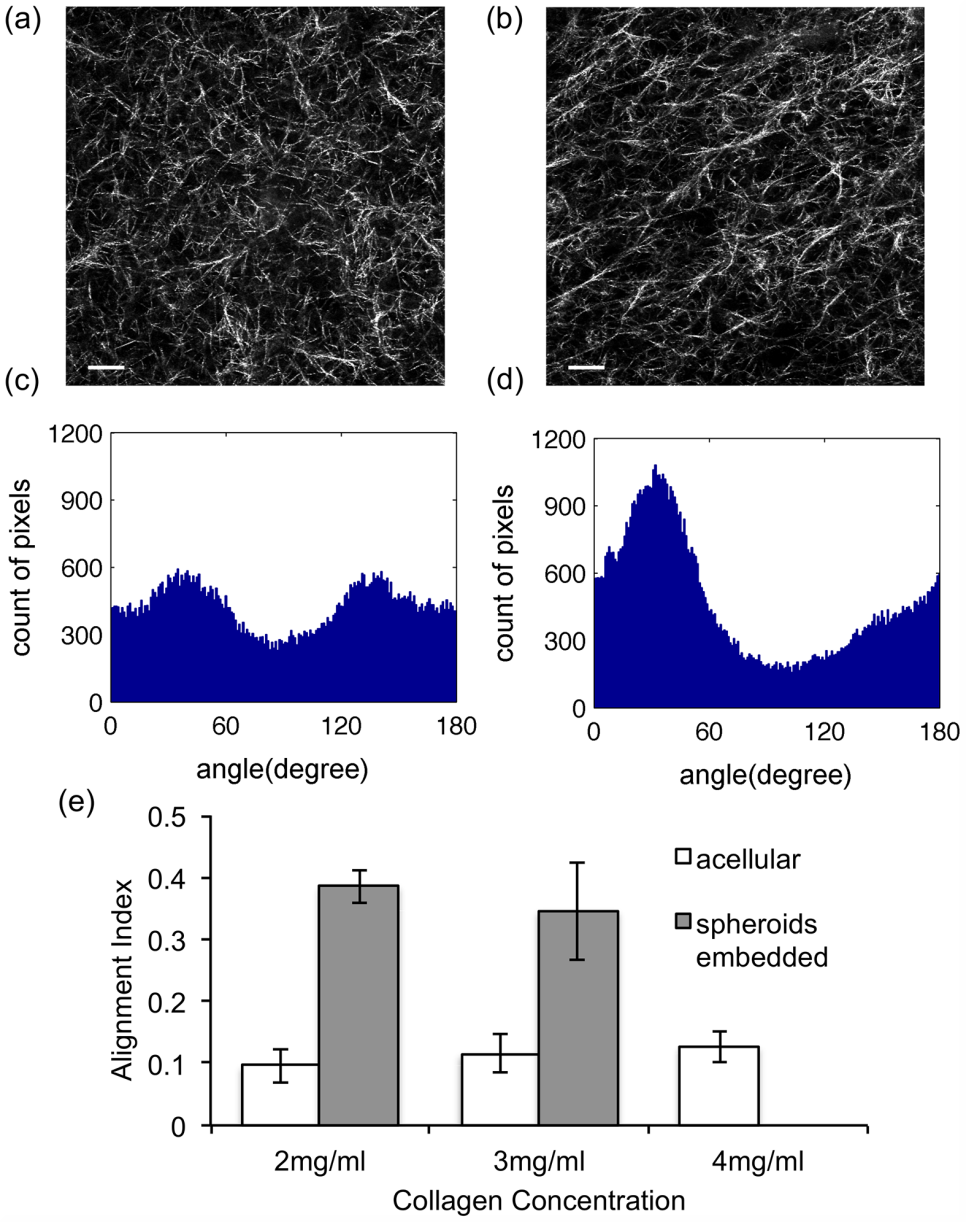
Alignment analysis of collagen gels at 3 mg/ml and comparison with 2 mg/ml. Images of acellular (a) and spheroid embedded (b) 3 mg/ml collagen gels were analyzed using the orientation detection algorithm. The scale bars are 20 *μm*. AI values were determined from the histograms of acellular collagen gels (c), AI = 0.086, and spheroid embedded collagen gels (d), AI = 0.357. (e) Among all the experimental collagen gel images, the AIs of acellular collagen gels are 0.096±0.027 at 2 mg/ml, 0.115±0.031 at 3 mg/ml, and 0.127±0.025 at 4 mg/ml. The AIs of 2mg/ml and 3mg/ml spheroid embedded collagen gel are 0.386±0.027 and 0.346±0.079. The error bars are the standard deviation calculated among all images under the same experimental conditions. They are all significantly different with each other by the Welch two sample t-test.

After validating the alignment quantification algorithm with the computer-generated images, the collagen fiber alignments of acellular and spheroid embedded collagen gels were characterized. Upon visual inspection, the fibers in acellular 2 mg/ml collagen gels were randomly aligned (Fig 3A) while those of the spheroid embedded 2 mg/ml collagen gels were aligned (Fig 3B). The histogram of the spheroid embedded gel (Fig 3D) had a well-defined symmetrical peak while that of the acellular gel (Fig 3C) did not, supporting the visual observation. Similar alignment patterns to the 2 mg/ml gels were seen in 3 mg/ml gels though expected increases in number of fibers were detected (Fig 4).

To quantify the degree of alignment, the AI was computed from the fiber orientation estimations obtained using the orientation detection algorithm. There are several features of alignment indicated by this quantitative result. First of all, with relatively smaller AI values, the acellular collagen gels are easily distinguishable from the spheroid embedded gels with larger AI values. Second, the difference in degree of alignment among the different concentrations of acellular collagen networks is also statistically significant as tested by t-test (P < 0.05). Third, the degree of collagen fiber alignment in spheroid embedded collagen gels (AI ~ 0.4) was less than the degree of alignment obtained by external strains (up to AI ~ 0.7) previously reported [16]. This difference is likely due to the contractile forces by cell clusters not being as large as the maximum external strain applied to the collagen gels.

To easily estimate the AI, an alternative method utilizing only the standard deviation of the pixel orientations was discovered. We tested the influence of both Gaussian and Von Mises distributions, fiber fraction, and fiber directionality on the relationship between AI and standard deviation and found no differences (data not shown). The AI only measures the degree of alignment, independent of directionality, distribution pattern, and data size of orientation. The AI is a reflection of the standard deviation thus it is possible to use the standard deviation to estimate the AI directly. This one-to-one relationship between the AI and the standard deviation of pixel orientation was obtained quantitatively using the data previously acquired from the simulated images. Using this one-to-one relationship, the AI of collagen fiber networks was estimated directly from the standard deviation. The error of this estimation method (~ 0.02 ± 0.01) was very small, providing an easier way of estimating the degree of alignment quantitatively and accurately.

**Fig 5:**
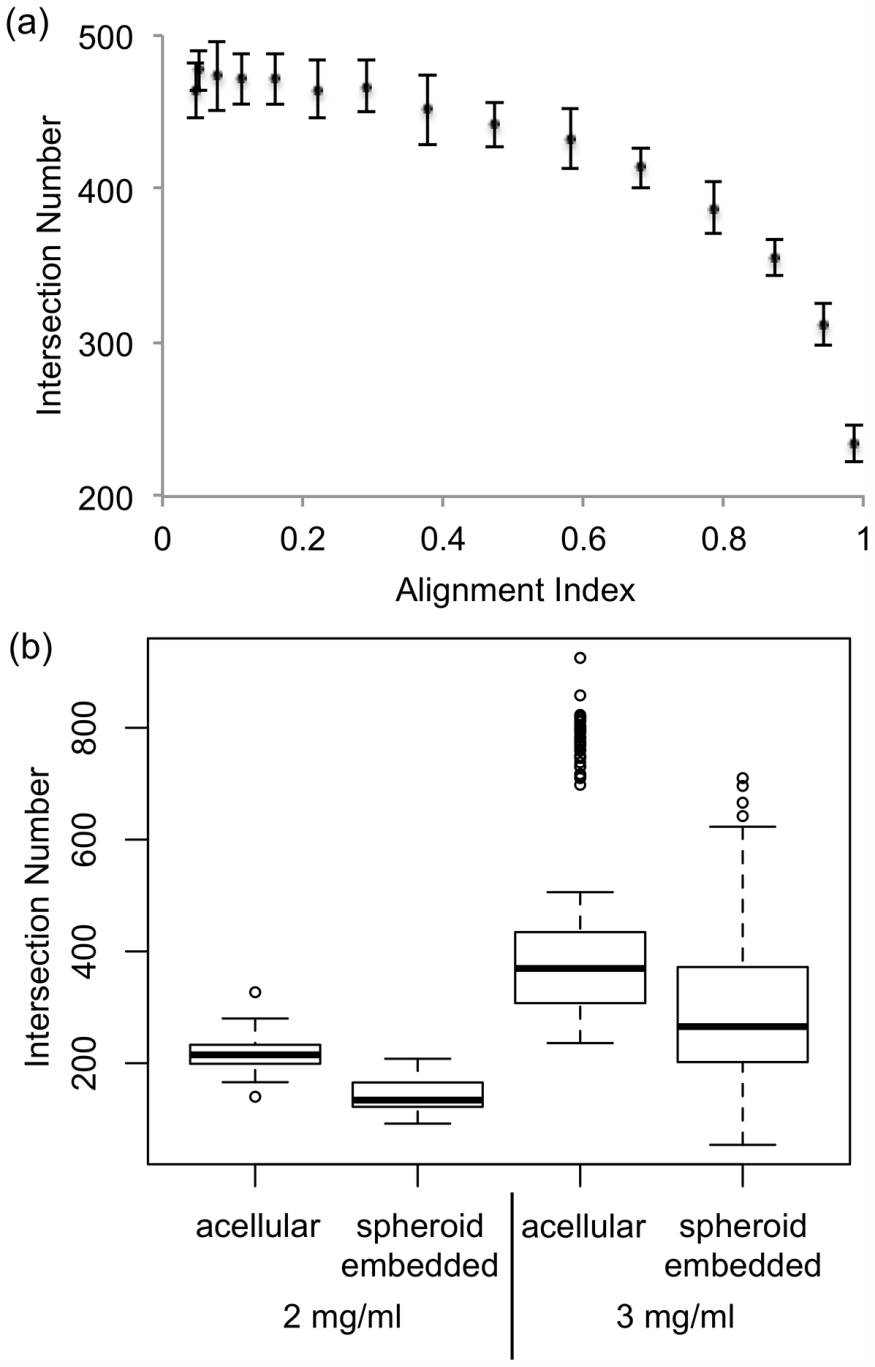
The number of fiber intersections decreases with increasing degree of alignment. Computer-generated fiber networks validated the intersection detection algorithm (a) and identified a general relationship between degree of alignment and intersection number geometrically. More intersections are present in networks when the AI is smaller. Using the ANOVA test, the correlation between the AI and the intersection number was significant with P < 0.05. (b) The number of fiber intersections in acellular gels was shown to be significantly larger than the one in the spheroid embedded collagen gels at the same collagen concentration (P < 0.05) using the Welch two sample t-test.

To explore additional applications for our method of quantifying fiber alignment, we further studied how the degree of fiber alignment affects the number of collagen fiber intersections. The intersection quantification method was validated with average errors around 5% at both 2mg/ml and 3mg/ml, using the same set of artificial images in validation of alignment algorithm. Manual counts of fiber intersections of the *in vitro* collagen gels were also used to reflect the error of the algorithm, which was determined to be within 15% on average, calculated by the percentage error between manual count and algorithm estimated results. The manual counts were performed by visually counting fiber intersections on 20 50×50 pixels *in vitro* collagen images, within which were 5 randomly segmented images per condition: acellular collagen gel at 2mg/ml and at 3 mg/ml, spheroid embedded gel at 2mg/ml and 3mg/ml. A negative correlation between the AI and fiber intersection number was discovered by analysis of the artificial images and supported with the intersection quantification results of the *in vitro* collagen gels (Fig 5).

The two components influencing collagen fiber intersections are the degree of alignment of the fibers and the fiber fraction. The fiber fractions of spheroid embedded gels, 12.8%±1.8% at 2 mg/ml and 20.7%±6.6% at 3 mg/ml, were relatively lower than the ones of acellular collagen gels, 15.9%±1.1% at 2 mg/ml and 26.7%±6.0% at 3 mg/ml. To investigate the correlation between alignment and the number of intersections, it is critical to account for change in intersections due to changes in fiber fraction using maximum likelihood multi-linear regression method (MLMLR) was used.

$$N_{intersection}=\beta_{0}+\beta_{1}AI+\beta_{2}fiber\%+\varepsilon ,$$

here *N*_*intersection*_ is the number of collagen fiber intersections, *AI* is the alignment index quantifying the degree of collagen fiber alignment, *fiber*% is the collagen fiber fraction, *β*_*i*_ is the regression or the correlation coefficient, i = 0, 1 or 2, and *ε* is the residuals or the error difference from the regression model. The 95% confidence intervals of each regression coefficients are: *β*_0_ ~ (−92.4, −80.7), *β*_1_~ (−45.0, −11.3) and *β*_2_ ~ (18.4, 18.9). All of these correlations were determined to be significant through ANOVA test (P < 0.05). The residuals of the regression model (*ε*) had a mean of zero and were uncorrelated with both AI and fiber fraction as tested individually in residual plots, confirming that the regression model is efficient. As the range of the 95% confidence interval of the AI’s regression coefficient was small and the entirety of the interval was less than zero, the MLMLR test showed a significant negative correlation between AI and intersection number.

## Discussion

The goal of this study is to provide both an accurate and computationally efficient method for quantifying the collagen fiber alignment at a pixel-wise level. Using both computational and experimental data, we are able to validate our method. This validation work extends the work by Daniels et al. [21] and Vader at al. [28], by also incorporating ‘fibers’ width, length distribution and, most importantly, varying degree of alignment of ‘fibers’. As a result, our algorithm provides information on different alignment patterns as well as a platform for collagen fiber feature detection in scenarios where there is a very large data sample and canonical statistical tests are mostly not available. We also extend the study to investigate the alignment features of collagen fibers aligned by the cells *in vitro*. The method of separately studying two entangled factors using the most likelihood multi-linear regression and the ANOVA provides the relationship between fiber alignment and intersection number. This paper also develops an easy and accurate estimate for AI values based on the standard deviation of pixel-wise orientation. Provenzano et al. [18] quantified the standard deviation of fiber alignments in the intratumoral, juxtatumoral and extratumoral regions of breast cancer patients and our accurate estimate for AI values would be useful in such studies. Our rapid algorithm in quantifying the pixel-wise fiber orientations and their degree of alignment could potentially be applied to *in vivo* cancer studies to quickly obtain the degree of collagen fiber alignment from a tissue biopsy.

Potentially there are several further applications for this gradient based pixel-wise orientation analysis. ECM reorganization during cell migration or by cell traction sometimes results in only local fiber remodeling [50], which would require a pixel-wise analysis of individual fibers or fiber fragments. The secondary orientation information, *dθ*_*Fiber Pixel*_ / *dt*, provides the fiber dynamics that global orientation algorithms, such as FFT, fail to detect. Beyond analysis of fibers, this algorithm could be utilized to detect cell membranes curvature and curvature change along membranes, *dθ*_*Membrane Pixel*_ / *dl*, within dynamic collective cell clusters [51–53].

Here, we also note several important features and limitations of our results that need further clarification. Firstly, the multi-linear regression model investigates actual collagen fibers with AI ~ [0, 0.45], within which a simple linear negative correlation should be sufficient to capture the intersection decrease due to AI. Whether this quantitative relationship can be further applied to larger range of AI requires additional sampling of actual collagen gels with higher degrees of alignment. Secondly, the intersection decrease with increasing AI values is less pronounced in the range of AI ~ [0, 0.6] among the generated images. This decrease in sensitivity of intersection detection, as well as the increased number of intersections compared to the actual collagen gels, may be due to the use of linear “fibers” for the generated images. Lastly, confocal reflectance microscopy has been noted to not visualize fibers that are nearly perpendicular to the focal plane due to Mie scattering [54,55]. This limitation leads to an inability to detect all of the fiber intersections within a 3D volume. Using either fluorescence confocal or multi-photon microscopy would eliminate this limitation.

Our work also aims to provide information detailing collagen alignment features. One feature is the correlation between collagen gel density and the AI identified within the acellular collagen gels. A mechanism that could lead to this observation has not previously been established. We hypothesize that it may be due to the rates of nucleation and elongation of the collagen fibers [56]. Another feature is the correlation between the intersections of collagen fibers and their degree of alignment. In simulations of fiber networks fiber crosslinks are usually identical to fibers intersections [12,57–59] or as a fraction of the number of intersections [60]. Crosslinking of collagen fibers have many biological applications, affecting collagen gel elastic modulus [61] and potentially inhibiting cell migration, as previous simulations reveal a possible mechanism for increased fiber crosslinks inhibition of cell migration [60]. Additionally, within highly aligned collagen fiber networks, cells migrate more rapidly due to fewer obstacles [16,62]. We believe that our approach may create avenues for further studies to elucidate the relationships between collagen fiber intersections, degree of total fiber crosslinking and cell migration and analyze the effects of drugs used to increase crosslinks within collagen gels [63–65], to provide a more complete picture of how the ECM structure determines cell migration.

## Conclusion

To quantitatively elucidate the physical features of collagen fiber alignment, we have developed a fast and sensitive quantification method and validated our results in an integrated computational and experimental study. By studying *in silico* fibers, *in vitro* gels and fibers around spheroids, we were able to determine a fast estimation method of alignment index *in vivo* using only the orientation standard deviation. These quantitative methods can be useful tools in large data analysis, especially in ECM reorganization during tumor progression. The correlation between the degree of alignment and the intersection number may play an important role in investigating the mechanism of how collagen fiber alignment effects cell migration and invasion, particularly during some of the most critical stages of tumor development and metastasis.
