# Correction: Rapid Quantification of 3D Collagen Fiber Alignment and Fiber Intersection Correlations with High Sensitivity

**DOI:** 10.1371/journal.pone.0157379

**Published:** 2016-06-07

**Authors:** Meng Sun, Alexander B. Bloom, Muhammad H. Zaman

In the version of this article initially published, the authors acknowledged the research support from the National Institutes of Health (U01-CA177799) and the National Science Foundation (DMR-1206335) for this work. The authors would like to also acknowledge the research support from the National Institutes of Health (P01HL120839).

## References

[1] Sun M, Bloom AB, Zaman MH (2015) Rapid Quantification of 3D Collagen Fiber Alignment and Fiber Intersection Correlations with High Sensitivity. PLoS ONE 10(7): e0131814 doi:10.1371/journal.pone.0131814 26158674 10.1371/journal.pone.0131814 PMC4497681

